# Supplementary material for: Multifactorial seroprofiling dissects the contribution of pre-existing human coronaviruses responses to SARS-CoV-2 immunity
Source: Nat Commun. 2021 Nov 18;12:6703. doi: 10.1038/s41467-021-27040-x (PMC8602384; doi:10.1038/s41467-021-27040-x)
Supplement: Supplementary file 1 — Supplementary Information [file 41467_2021_27040_MOESM1_ESM.pdf]

Multifactorial seroprofiling dissects the contribution of pre-existing human coronaviruses responses to SARS-CoV-2 immunity

Irene A. Abela[1,2$], Chloé Pasin[1,2$], Magdalena Schwarzmüller[1$], Selina Epp[1], Michèle E. Sickmann[1], Merle M. Schanz[1], Peter Rusert[1], Jacqueline Weber[1], Stefan Schmutz[1], Annette Audigé[1], Liridona Maliqi[1], Annika Hunziker[1], Maria C. Hesselman[1], Cyrille R. Niklaus[1], Jochen Gottschalk[3], Eméry Schindler[3], Alexander Wepf[4], Urs Karrer[5], Aline Wolfensberger[2], Silvana K. Rampini[6], Patrick M. Meyer Sauteur[7], Christoph Berger[7], Michael Huber[1], Jürg Böni[1], Dominique L. Braun[1,2], Maddalena Marconato[8], Markus G. Manz[8], Beat M. Frey[3], Huldrych F. Günthard[1,2*], Roger D. Kouyos[1,2*], Alexandra Trkola[1*]

[$]Equally contributing authors

[*]Jointly supervising authors

Affiliations

[1] Institute of Medical Virology, University of Zurich, Switzerland

[2] Division of Infectious Diseases and Hospital Epidemiology, University Hospital Zurich, Switzerland

[3] Blood Transfusion Service Zurich, Switzerland

[4] Institute of Laboratory Medicine, Cantonal Hospital Winterthur, Switzerland

[5] Department of Medicine, Cantonal Hospital Winterthur, Switzerland

[6] Department of Internal Medicine, University Hospital Zurich, Switzerland

[7] Division of Infectious Diseases and Hospital Epidemiology, University Children's Hospital Zurich, Switzerland

[8] Department of Medical Oncology and Hematology, University Hospital and University of Zurich, Switzerland

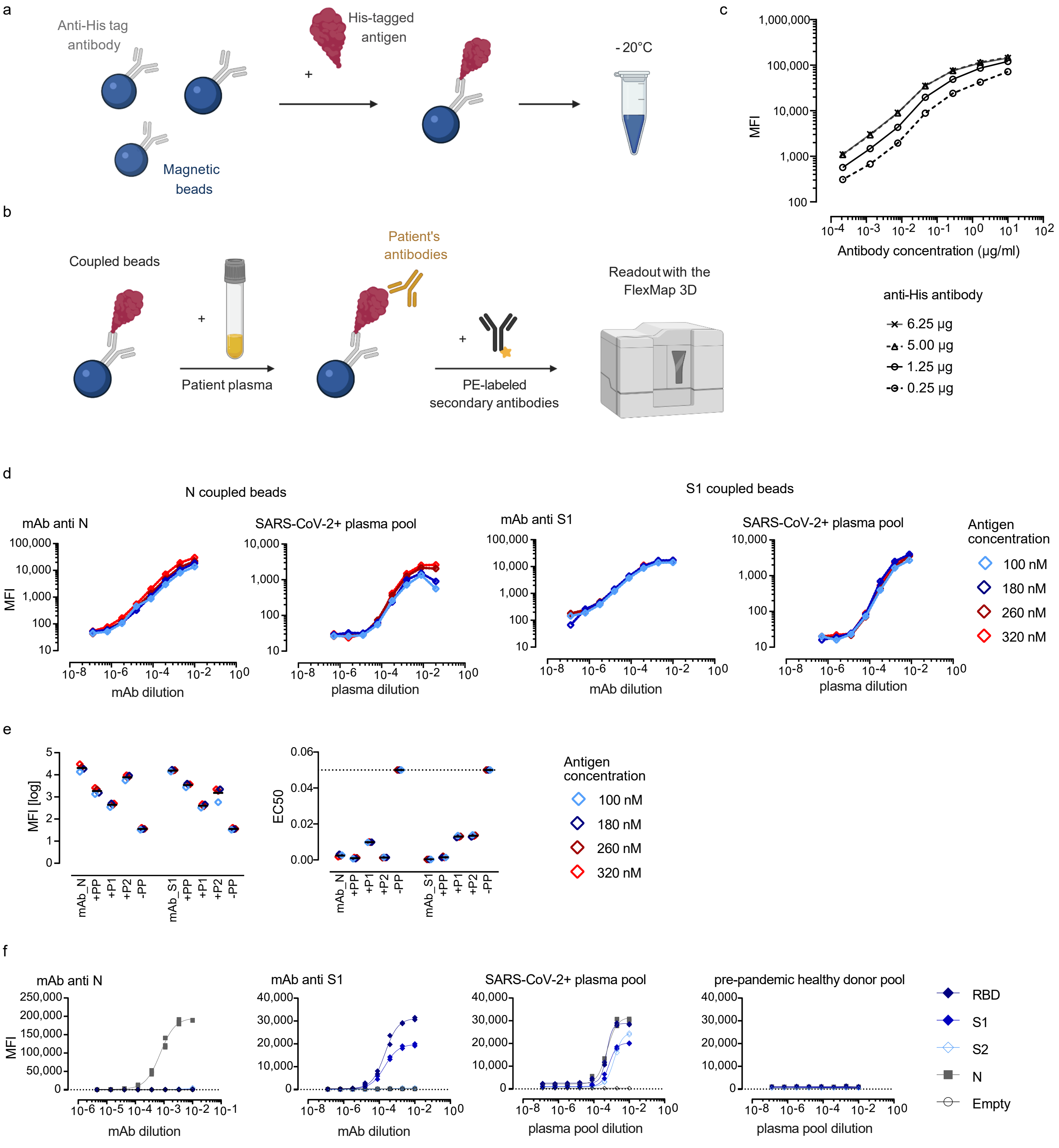

Supplementary Fig. 1. Establishment of ABCORA seroprofiling. (a) Directed coupling of His-tagged antigens to magnetic beads covalently coupled with anti-His antibody. (b) Binding of patient plasma antibodies to antigen-coupled beads and detection by PE-labeled secondary antibodies (IgG, IgA or IgM) with the FlexMap 3D reader (Luminex). Median fluorescence intensity (MFI) proportional to bound secondary antibody is recorded. Figure created with BioRender.com. (c) Titration of anti-His capture antibody on magnetic beads. One of two independent experiments is depicted. (d-e) Optimization of antigen loading. (d) Reactivity of beads loaded with increasing doses of SARS-CoV-2 nucleoprotein (N) or SARS-CoV-2 spike protein subunit S1 (S1) with titered anti-N and anti-S1 mAbs and a SARS-CoV-2 positive patient plasma pool. One of three independent experiments is depicted. (e) Median fluorescence intensity (MFI) at 1/100 dilution and the 50 % effective concentration (EC50) values for anti-N and anti-S1 mAbs, the SARS-CoV-2 positive patient pool (+PP), two individual SARS-CoV-2 positive patient plasma (P1 and P2) and a plasma pool of pre-pandemic healthy donors (-PP). One of two independent experiments is depicted. (f) Final assessment of assay setup (5 µg anti-His Ab per million beads, 320 nM His-tagged antigens, Phycoerythrin (PE)-labeled secondary antibodies at 17500). Reactivity of the indicated SARS-CoV-2 antigens with serial dilutions of anti-N and anti-S mAbs, positive and negative donor plasma pools was probed. At least two independent experiments are depicted.

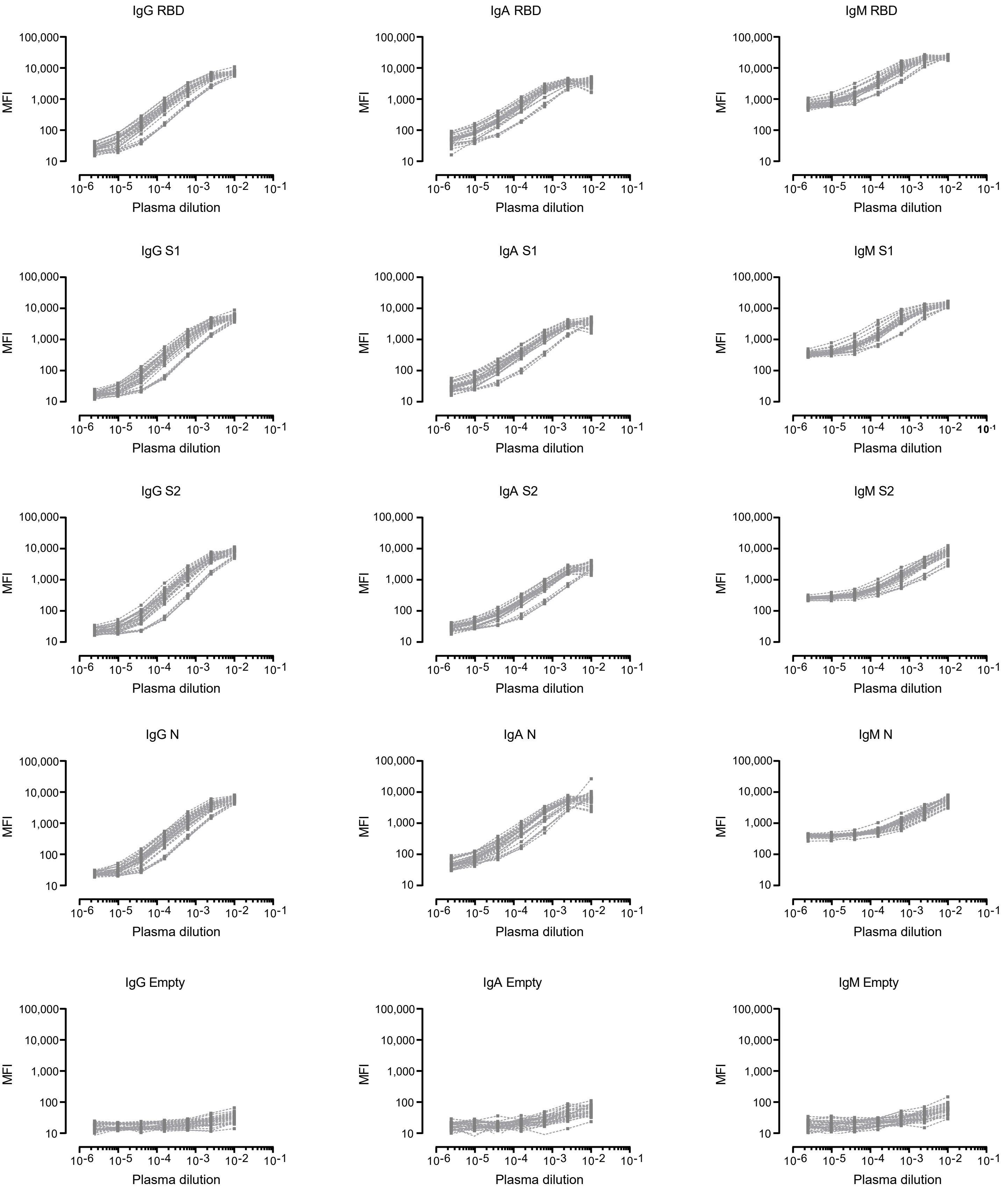

Supplementary Fig. 2. Assessment of assay variability. Titration of the positive control plasma donor pool composed of 20 SARS-CoV-2 RT-PCR positive patients. Median fluorescence intensity (MFI) for IgG, IgA and IgM reactivities to SARS-CoV-2 proteins (RBD, S1, S2, N) and empty bead reactivity of 31 independent titrations are shown.

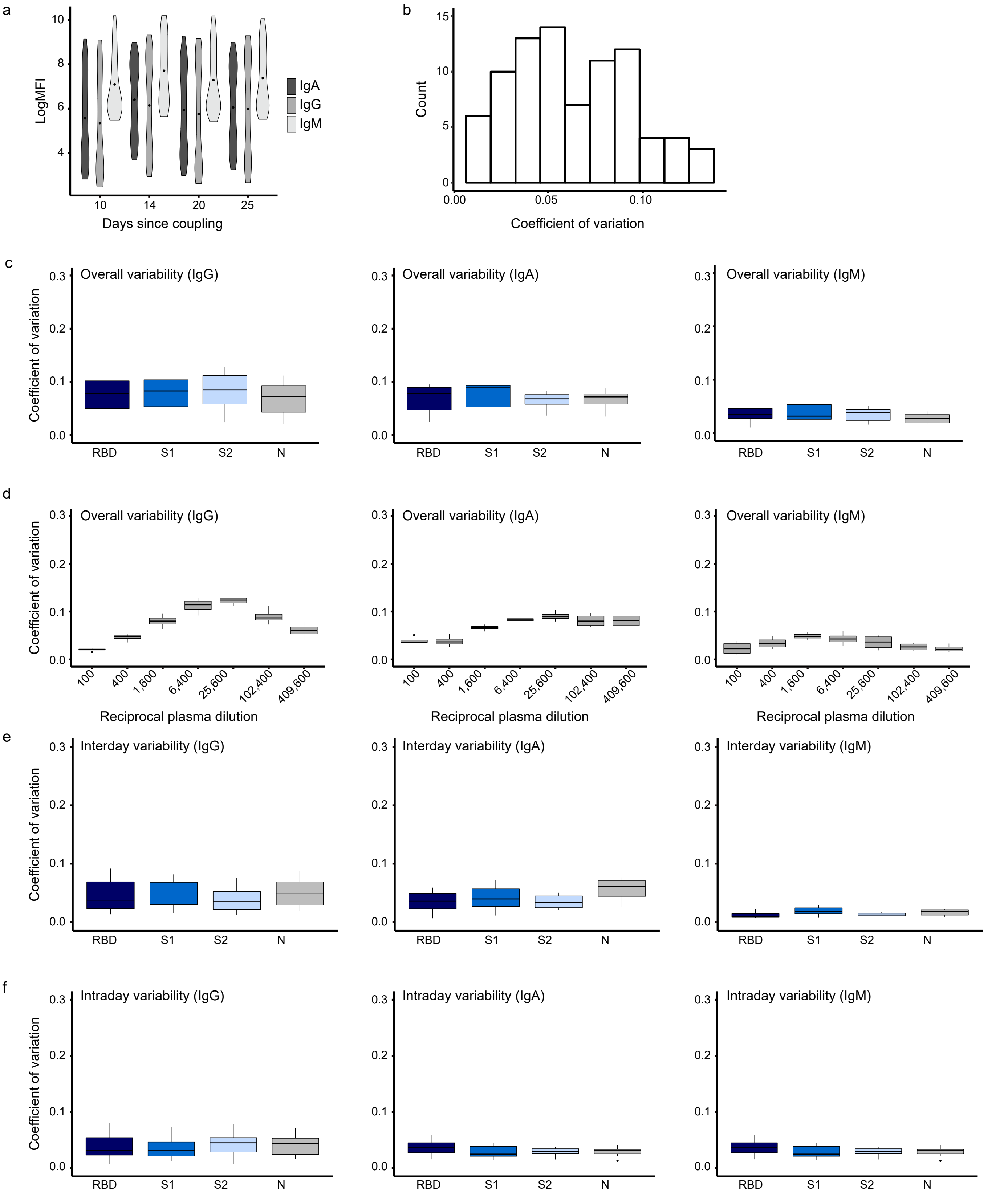

Supplementary Fig. 3. Temporal stability and variability analysis. (a) Assessment of the temporal stability of antigen coupled beads. The SARS-CoV-2 positive plasma pool was titrated on 25 days and the distribution of all signal intensities (pooled over plasma dilutions and antigens) for each day depicted. (b) Histogram of the overall assay variability (coefficient of variation) for all tested Ig classes based on the variability of mean log10 MFI values from 31 independent titrations (7 dilution steps) of the positive control plasma pool depicted in Supplementary Fig. 2. (c-d) Boxplots depicting the overall assay variability stratified by the four different antigens (c) and plasma dilutions (d) based on 31 independent titrations (7 dilution steps) of a positive plasma pool. (e) Boxplots showing the intraday variability stratified by the four different antigens based on six independent titrations of a positive plasma pool performed on the same day. (f) Boxplots showing the intraday variability stratified by the four different antigens based on a titration of a positive plasma pool performed on 10 different days. All boxplots represent the following: median with the middle line, upper and lower quartiles with the box limits, and 1.5x interquartile ranges with the whiskers.

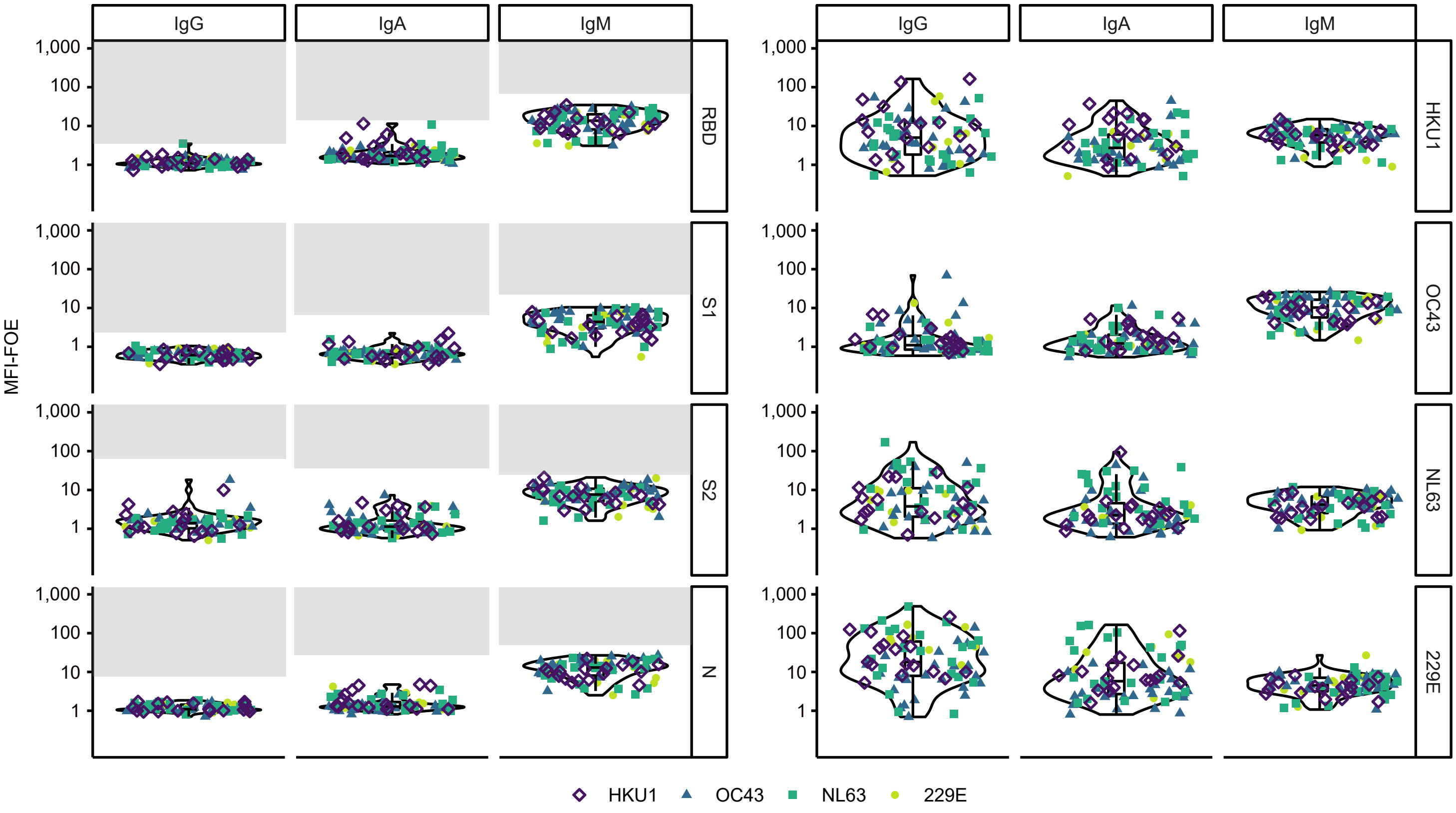

Supplementary Fig. 4. Training cohort II – recent HCoV infection. Assessment of the multiplex SARS-CoV-2 ABCORA 5.0 on the pre-pandemic individuals with recent HCoV infection (training cohort II). Depicted are MFI signals normalized to empty bead controls (MFI-FOE). Grey boxes indicate values above the individually set MFI-FOE cut-offs for SARS-CoV-2 specific responses for each antigen (see Supplementary Table 4). Each symbol and color corresponds to one HCoV (HKU1: N=17, OC43: N=27, NL63: N=22, 229E: N=9). Boxplots represent the following: median with the middle line, upper and lower quartiles with the box limits, and 1.5x interquartile ranges with the whiskers.

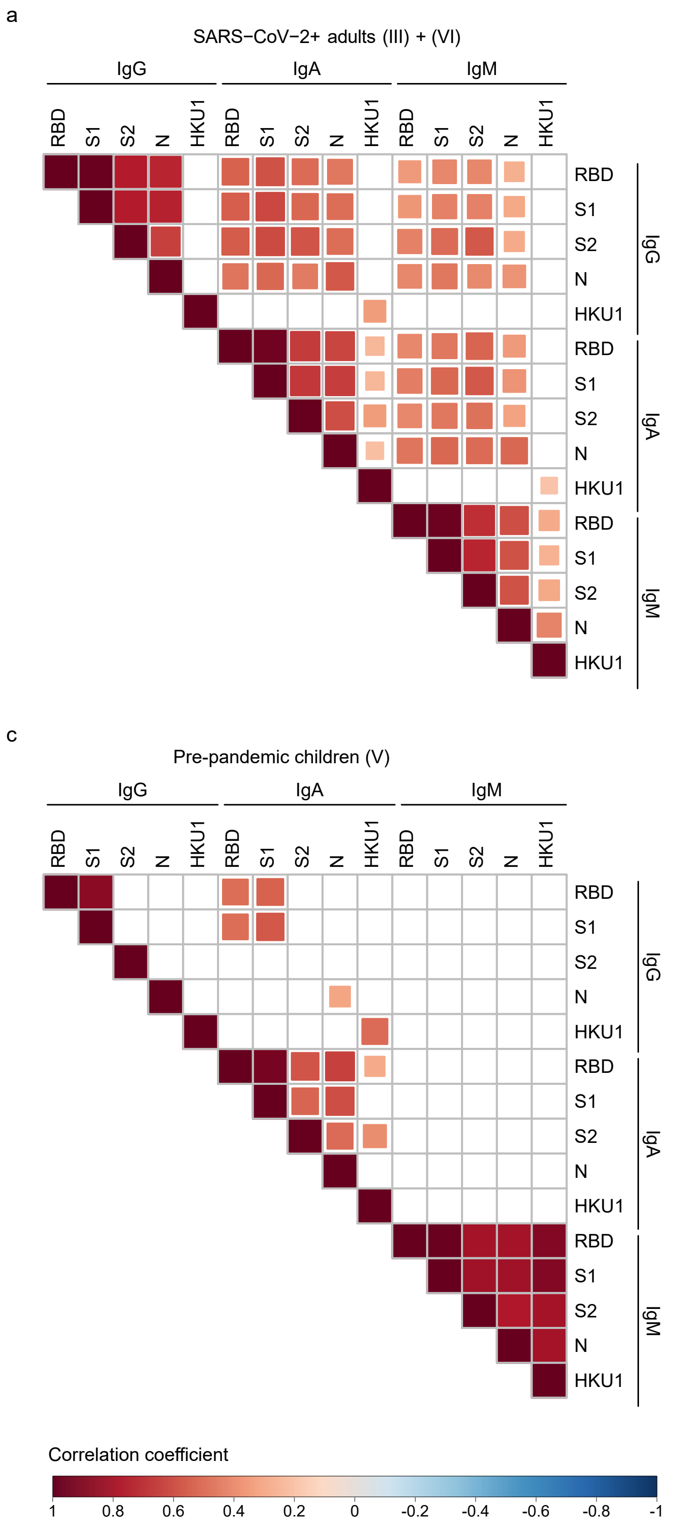

Supplementary Fig. 5. Interdependency of SARS-CoV-2 and HCoV HKU1 antibody reactivity. Spearman correlation matrix of SARS-CoV-2 (RBD, S1, S2, N) and HKU1 S1 antigen reactivity (based on logMFI-FOE) in (a) SARS-CoV-2 positive adults (N=389), (b) healthy, pre-pandemic adults (N=825), (c) pre-pandemic children (N=169) and (d) pre-pandemic samples from patients recently infected with a circulating HCoV strain (N=75). Non-significant correlations are left blank. Levels of significance are assessed by a two-sided test on the asymptotic t approximation of Spearman's rank correlation, and corrected by the Bonferroni method for multiple testing ($p<0.05/420$).

a

b

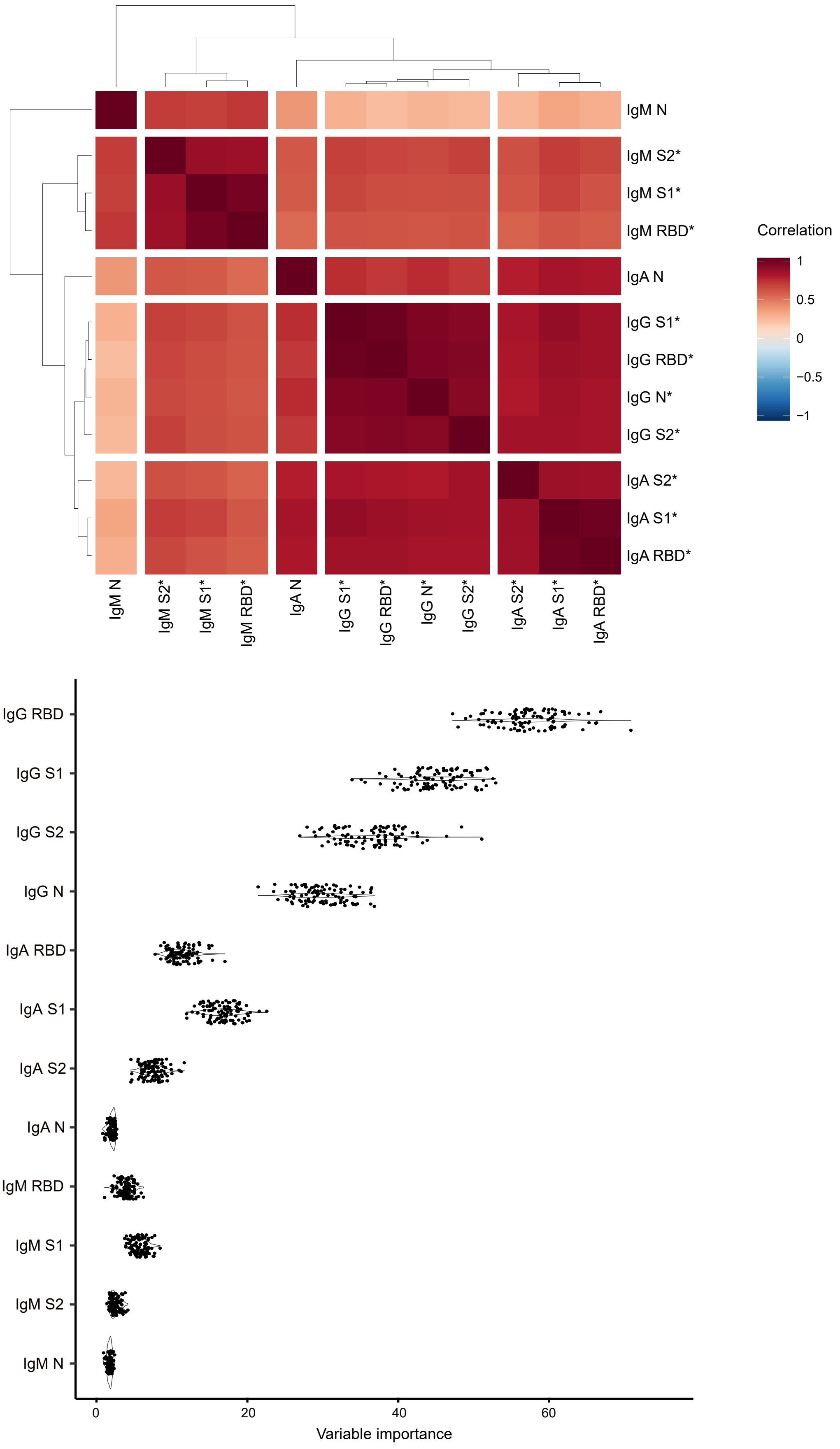

Supplementary Fig. 6. Variable importance for computational models. (a) Correlation matrix of all immunoglobulin variables in SARS-CoV-2 positive patients from the training dataset (N=175). Defining five clusters based on hierarchical clustering showed that IgA N and IgM N clustered separately from other IgA and IgM variables. Other variables (indicated by stars: all IgGs, IgAs without N and IgMs without N) were highly correlated. We therefore used the mean of these three clusters in the logistic regression. (b) Variable importance (measured as the mean decrease of node impurity with Gini index). Each of the 100 dots corresponds to a random forest performed on a bootstrap sample of the training dataset (N=823).

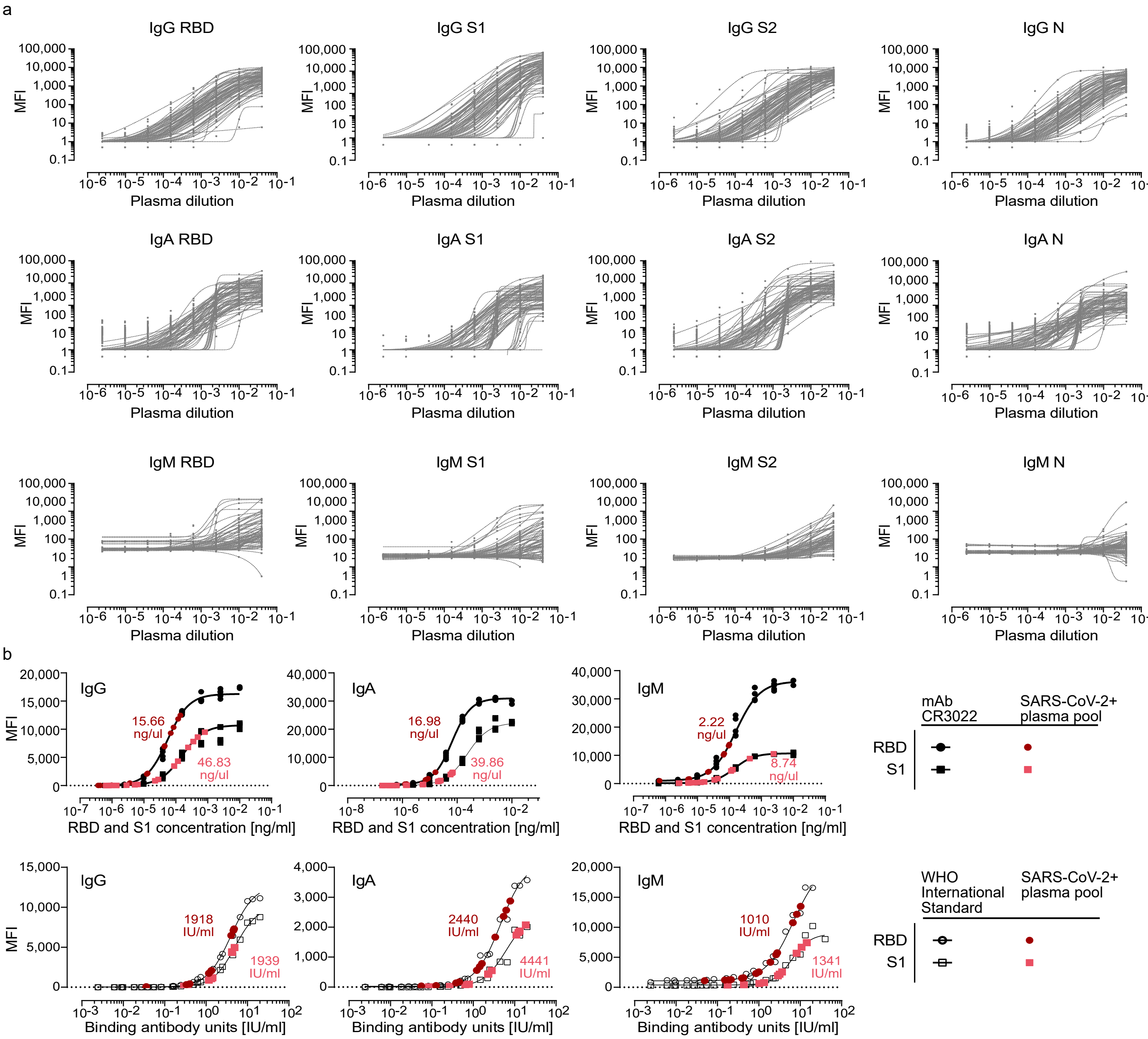

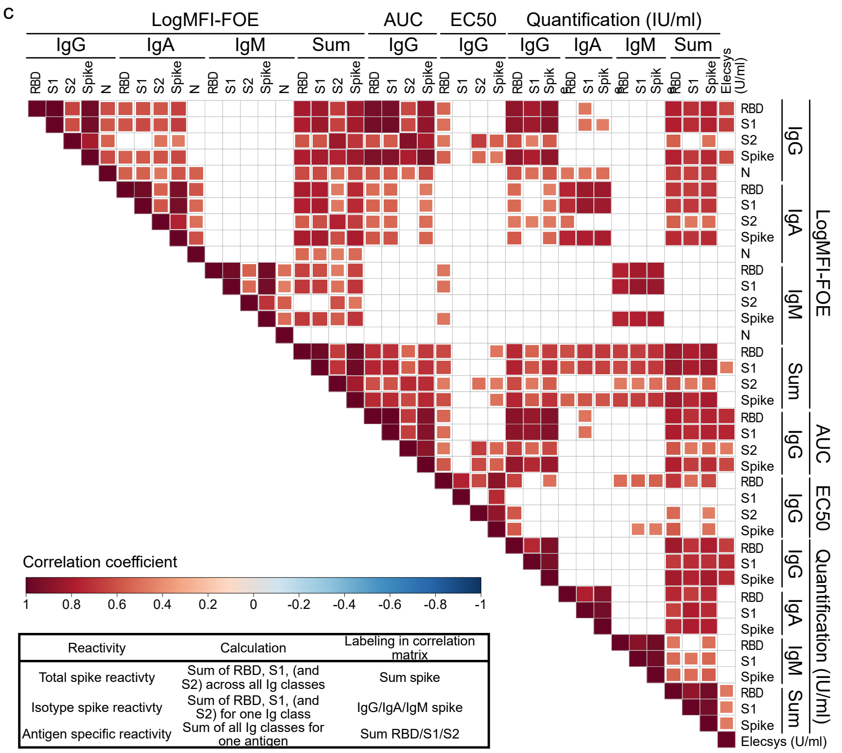

Supplementary Fig. 7. Quantification of SARS-CoV-2 specific antibodies. (a) Titration of SARS-CoV-2 positive plasma pool used as plate standard curve for quantification in Fig. 2 (N=72). Uncorrected MFI values are depicted. (b) Quantification of SARS-CoV-2 specific antibodies in the positive plasma pool by interpolating RBD and S1 content based on standard curves of the RBD-specific mAb CR3022 (upper panel) or the WHO International Standard (lower panel). Data from three independent experiments are depicted. (c) Spearman correlation matrix assessing agreement between diverse ABCORA measurements and quantifications (individual and summed logMFI-FOE values at 1/100 dilution of plasma, EC50, AUC, IU/ml content based on WHO Standard NIBSC 20/136) and Roche Elecsys Anti-SARS-CoV-2 (S) (U/ml). Non-significant correlations are left blank. Levels of significance are assessed by a two-sided test on the asymptotic t approximation of Spearman's rank correlation, and corrected by the Bonferroni method for multiple testing (p<0.05/780). Color shading denotes correlation coefficient.

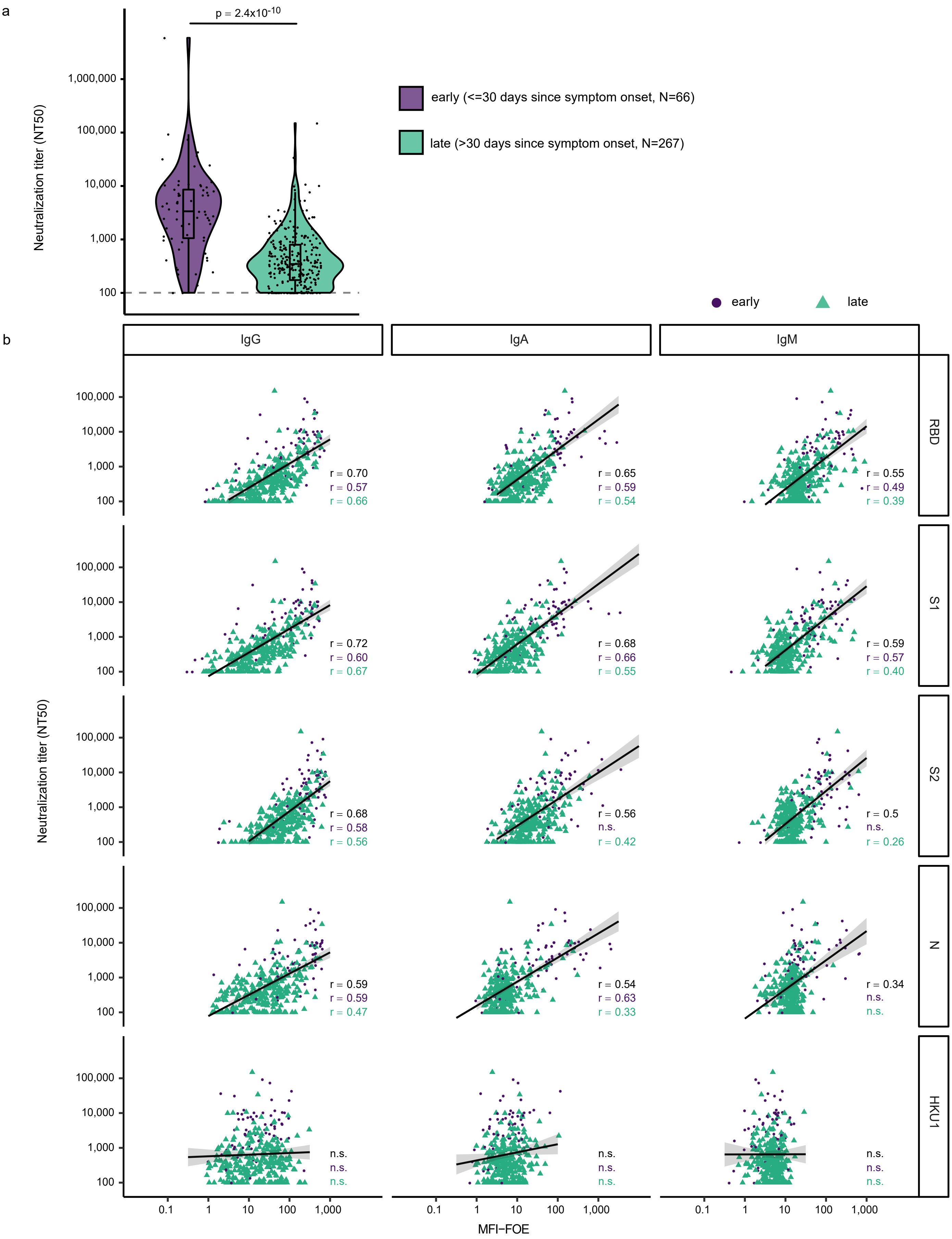

Supplementary Fig. 8. Association of binding and neutralization activity in early and late infection. (a) 50% Neutralization titers (NT50) titers against Wuhan-Hu-1 pseudotype in patients with known date of symptoms onset (N= 333). Patients were stratified according to time since first diagnosis to investigate early (less than 30 days post symptoms onset, lavender) and late (more than 30 days symptoms onset, turquoise) neutralization responses. Difference between these two groups was assessed with a linear mixed model with time since symptom onset (binary variable early/late) as fixed effect and individual as random effect and using a Satterthwaite approximation for a two-sided t-test on the parameter associated with time since symptom onset. (b) Linear regression analysis to define association between neutralization (reciprocal NT50) and antibody binding (MFI-FOE). Black lines indicate linear regression predictions. Levels of significance are assessed by a two-sided test on the asymptotic t approximation of Spearman's rank correlation, and corrected by the Bonferroni method for multiple testing (p<0.05/1200, see Supplementary Fig. 9).

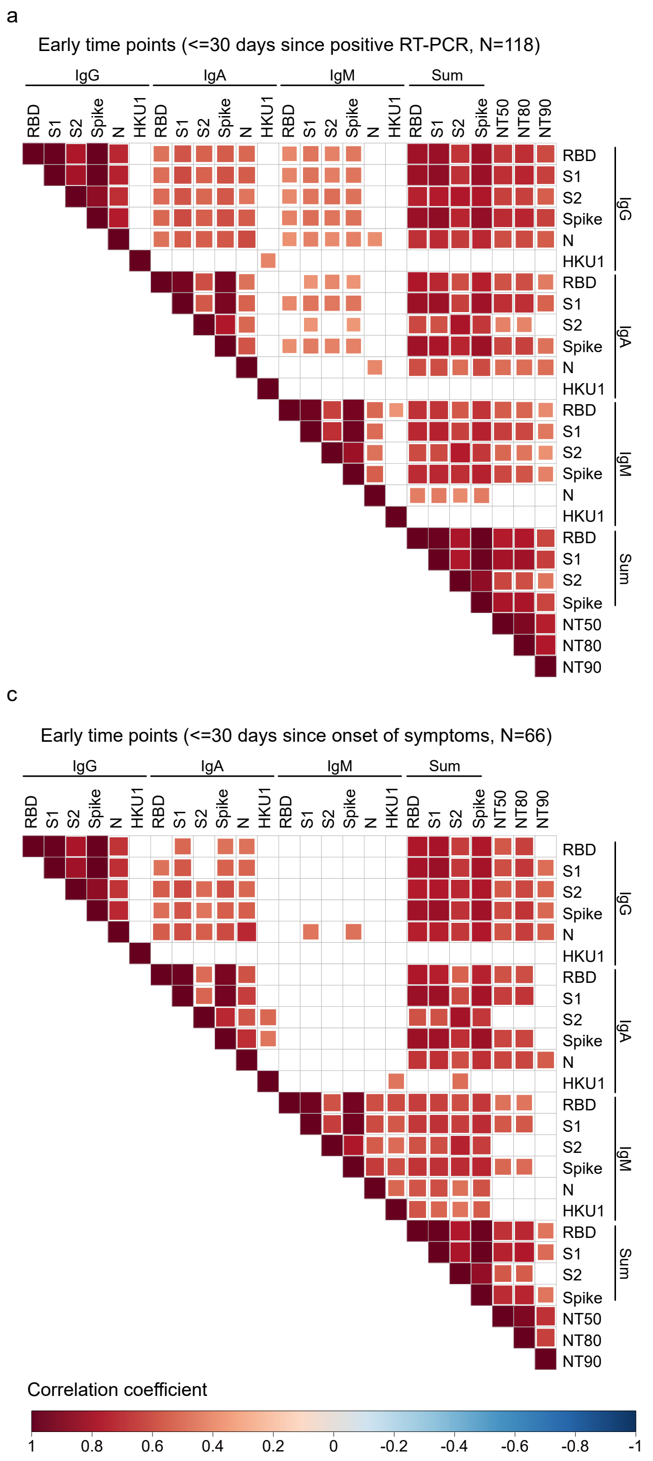

Supplementary Fig. 9. Correlation of antibody binding and neutralization activity in early and late infection. Spearman correlation matrix assessing agreement between SARS-CoV-2 antigen reactivity (RBD, S1, S2, N) based on logMFI-FOE values and neutralization (NT50, NT80, NT90) in SARS-CoV-2 positive adults (N= 389) divided in (a) early and (b) late time points corresponding to time since positive RT-PCR diagnosis (a, N=118 – b, N=251) or (c) early and (d) late corresponding to time since symptom onset (c, N=66 – d, N=267). Non-significant correlations are left blank. Levels of significance are assessed by a two-sided test on the asymptotic t approximation of Spearman's rank correlation, and corrected by the Bonferroni method for multiple testing (p<0.05/1200).

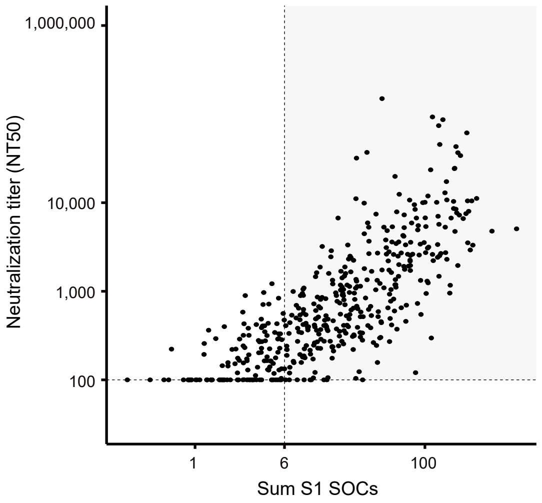

Supplementary Fig 10. Predicting neutralization capacity as a function of binding activity. Neutralization prediction based on a modified ULR-S1 model utilizing the diagnostic readout SOC instead of MFI-FOE values as input. Measured NT50 value versus sum of S1 SOC values (IgG, IgA, IgM) are depicted. Dashed lines correspond to a NT50=100 horizontally and the sum S1 SOCs=6 vertically. The sum S1 SOCs=6 corresponds to a specificity=84% and a sensitivity=80%. The grey shaded area corresponds to true positives (individuals with NT50 >100 predicted as neutralizers).

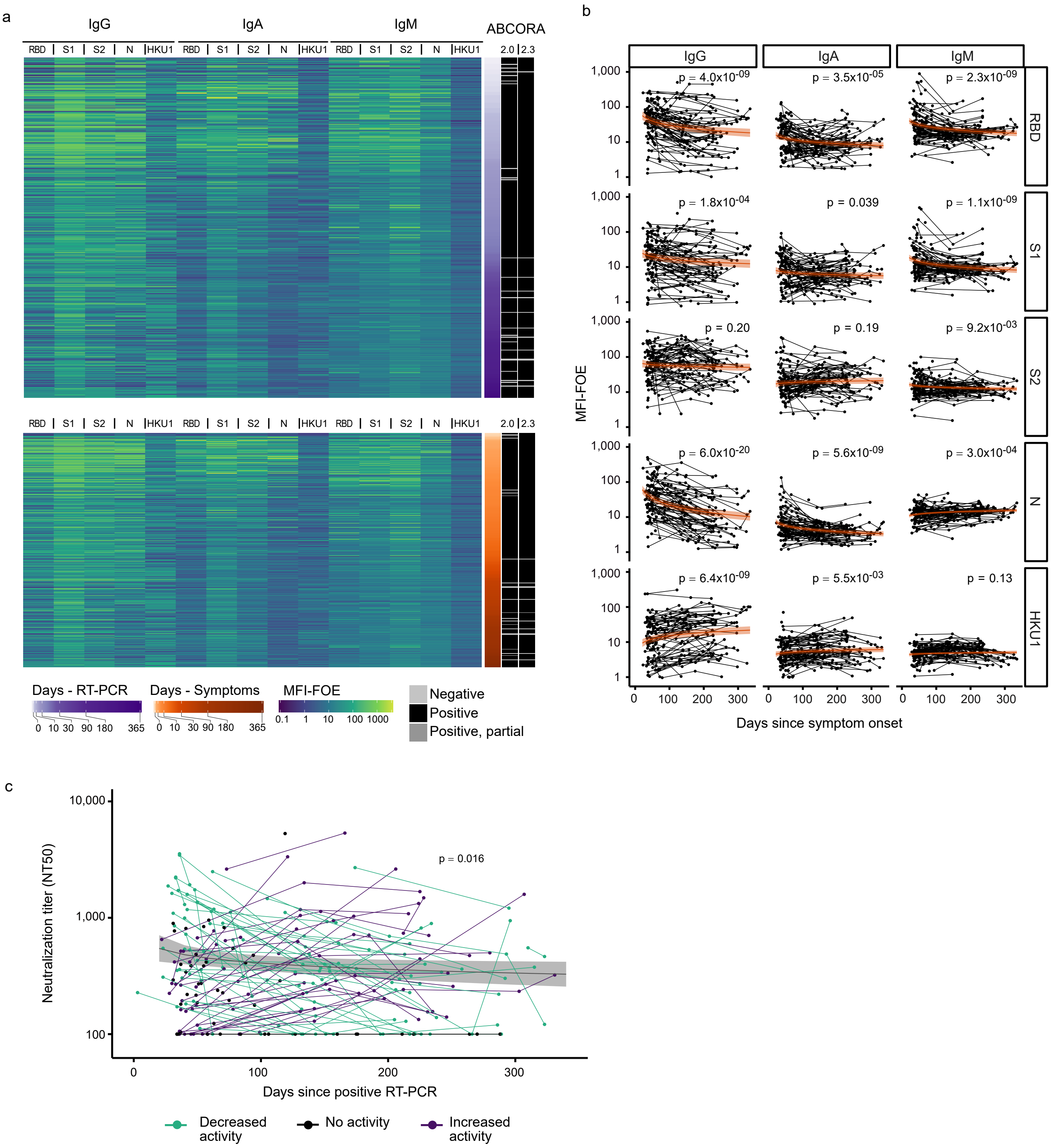

Supplementary Fig. 11. Monitoring temporal evolution of antibody responses (a) Heatmaps representing the measured MFI-FOE values and the outcome predicted with ABCORA 2.0 - 2.3 of measurements of SARS-CoV-2 positive patients with known dates of positive RT-PCR diagnosis (N=369) (upper panel) or with known dates of onset of symptoms (N=333) (lower panel). Purple and orange scales indicate days post positive RT-PCR or days post onset of symptoms, respectively, white-to-black scale indicates seroconversion predicted with the different ABCORA approaches. (b) Linear mixed model, with time since symptom onset as fixed effect and individual as random effect, estimating the decay of antibody binding activity based on ABCORA 2.0 measurements at 1-4 longitudinal time points in 120 individuals totaling in 251 measurements. Orange lines correspond to the models estimation and orange shaded areas to the 95% confidence intervals. Antibody half-lives (t1/2 in days) from significant models are depicted. Significance was assessed using Satterthwaite approximation for a two-sided t-test on the decay parameters. (c) Linear mixed model estimating the decay of neutralizing capacity in patients separated by their neutralizing activity. Only individuals with NT50>100 at their first measurement were used to estimate the half-life. The black line corresponds to the model estimation and the grey shaded area to the 95% confidence interval. Significance was assessed using Satterthwaite approximation for a two-sided t-test on the slope parameters.

a

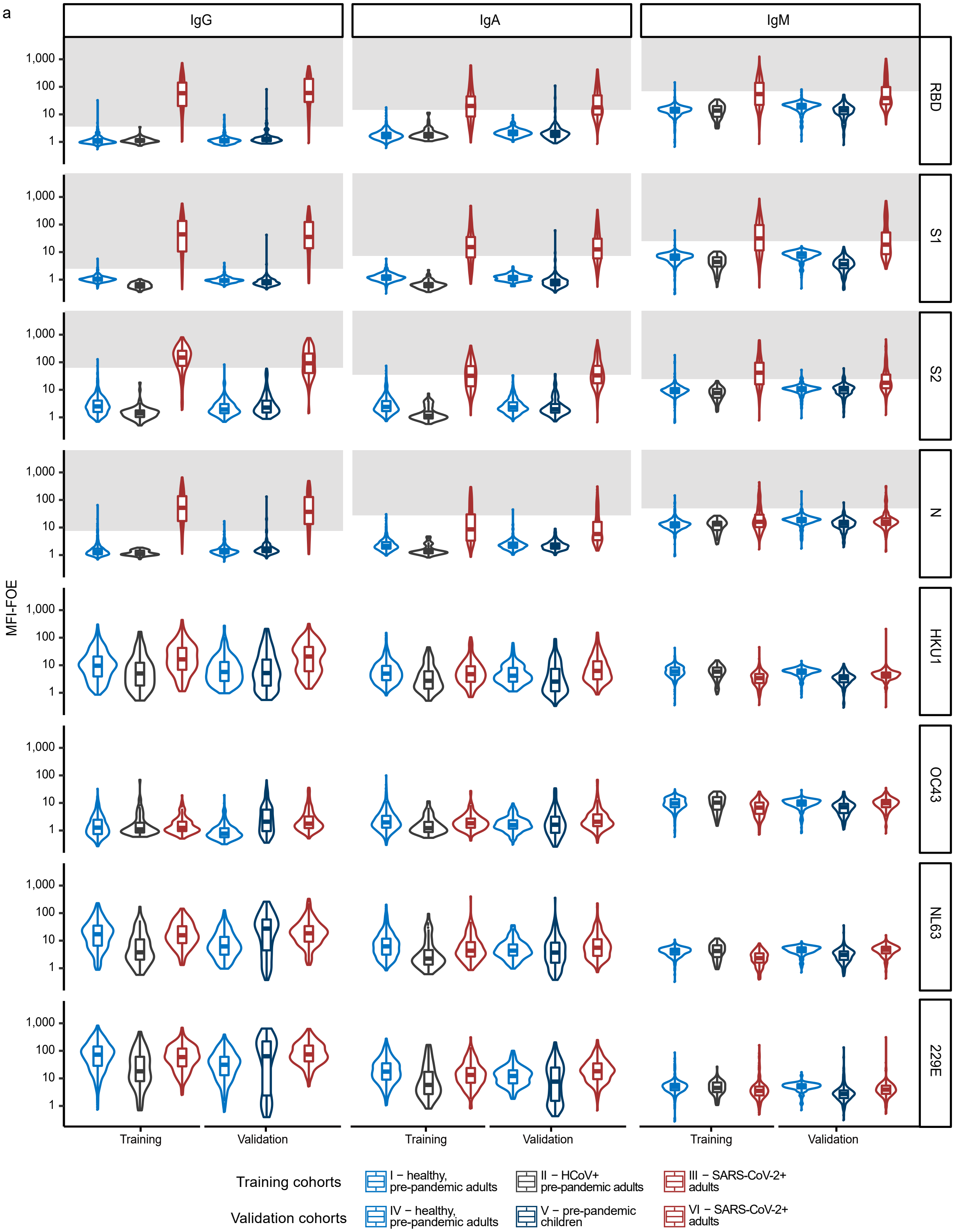

b

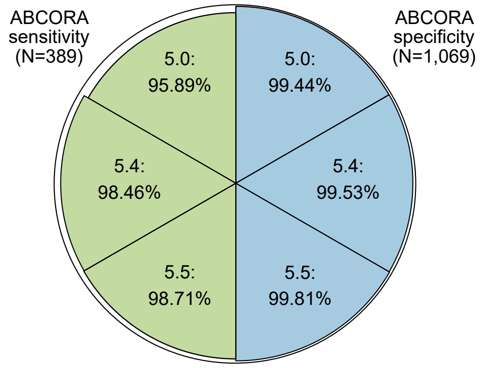

Supplementary Fig. 12. ABCORA 5 seroprofiling records antibody reactivity to SARS-CoV-2 and four HCoVs. (a) Assessment of the multiplex SARS-CoV-2 ABCORA 5.0 on the indicated training (N= 825) and validation (N=635) cohorts (Supplementary Table 3). Depicted are MFI signals normalized to empty bead controls (MFI-FOE). Grey boxes indicate values above the individually set MFI-FOE cut-offs for SARS-CoV-2 specific responses for each antigen (see Supplementary Table 4). Boxplots represent the following: median with the middle line, upper and lower quartiles with the box limits, and 1.5x interquartile ranges with the whiskers. (b) Sensitivity and specificity of ABCORA 5 assay versions 5.0, 5.4 and 5.5 based on the combined training and validation cohort data depicted in (a) (see also Supplementary Table 10). False negative proportion (sensitivity; green) and false positive proportion (specificity; blue) samples are represented by the reduction from 100% (outer circle) per segment.

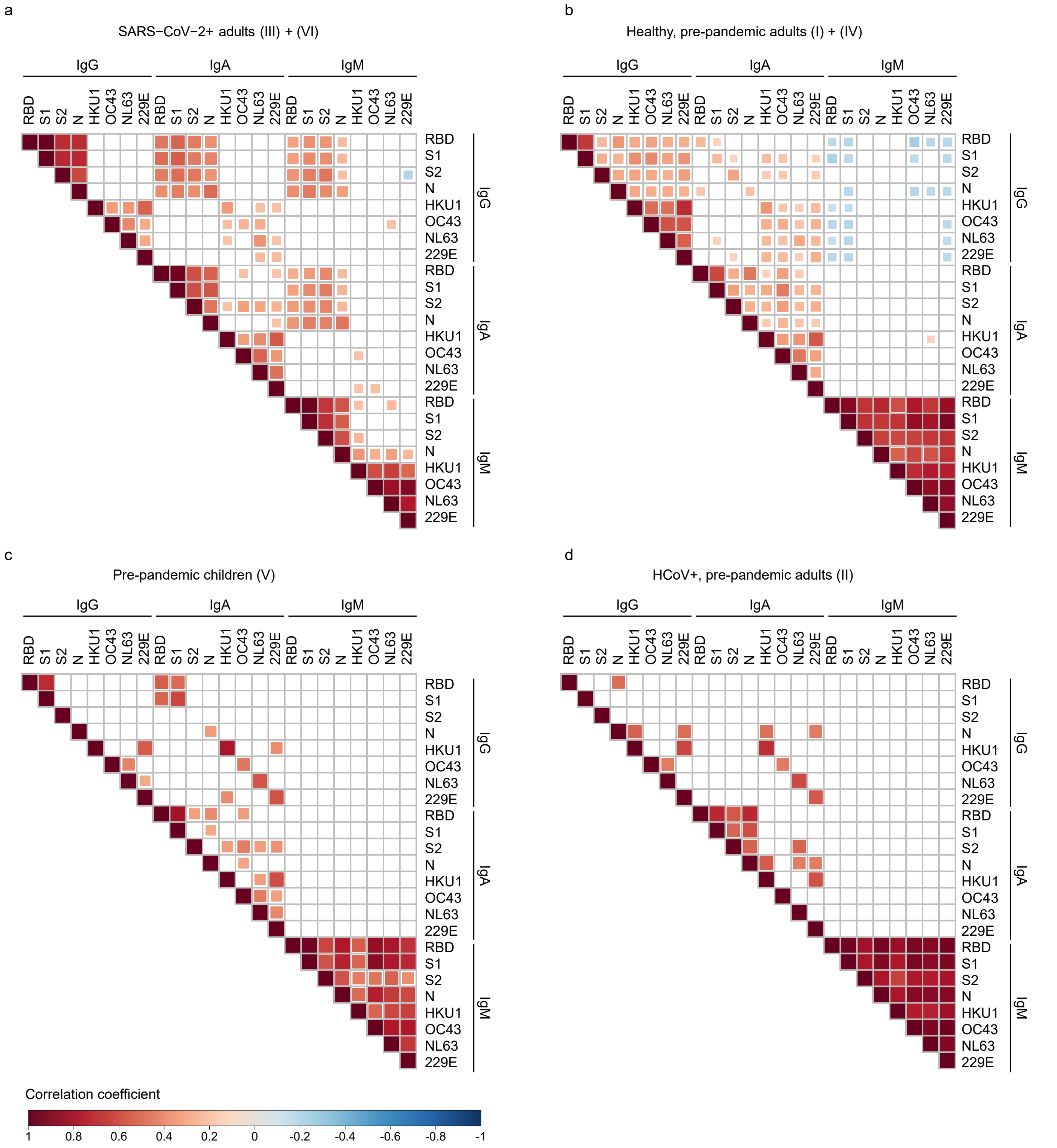

Supplementary Fig. 13. Interdependencies between antibody reactivity to SARS-CoV-2 and the four HCoVs. Spearman correlation matrix assessing agreement between SARS-CoV-2 antigens (RBD, S1, S2, N) and HCoVs (229E, NL63, OC43, HKU1) (based on logMFI-FOE) in (a) SARS-CoV-2 positive adults (N= 389), (b) healthy, pre-pandemic adults (N= 825), (c) pre-pandemic children (N=169) and (d) pre-pandemic samples from patients recently infected with a circulating HCoV strain (N=75). Non-significant correlations are left blank. Levels of significance are assessed by a two-sided test on the asymptotic t approximation of Spearman's rank correlation, and corrected by the Bonferroni method for multiple testing ($p<0.05/1104$).

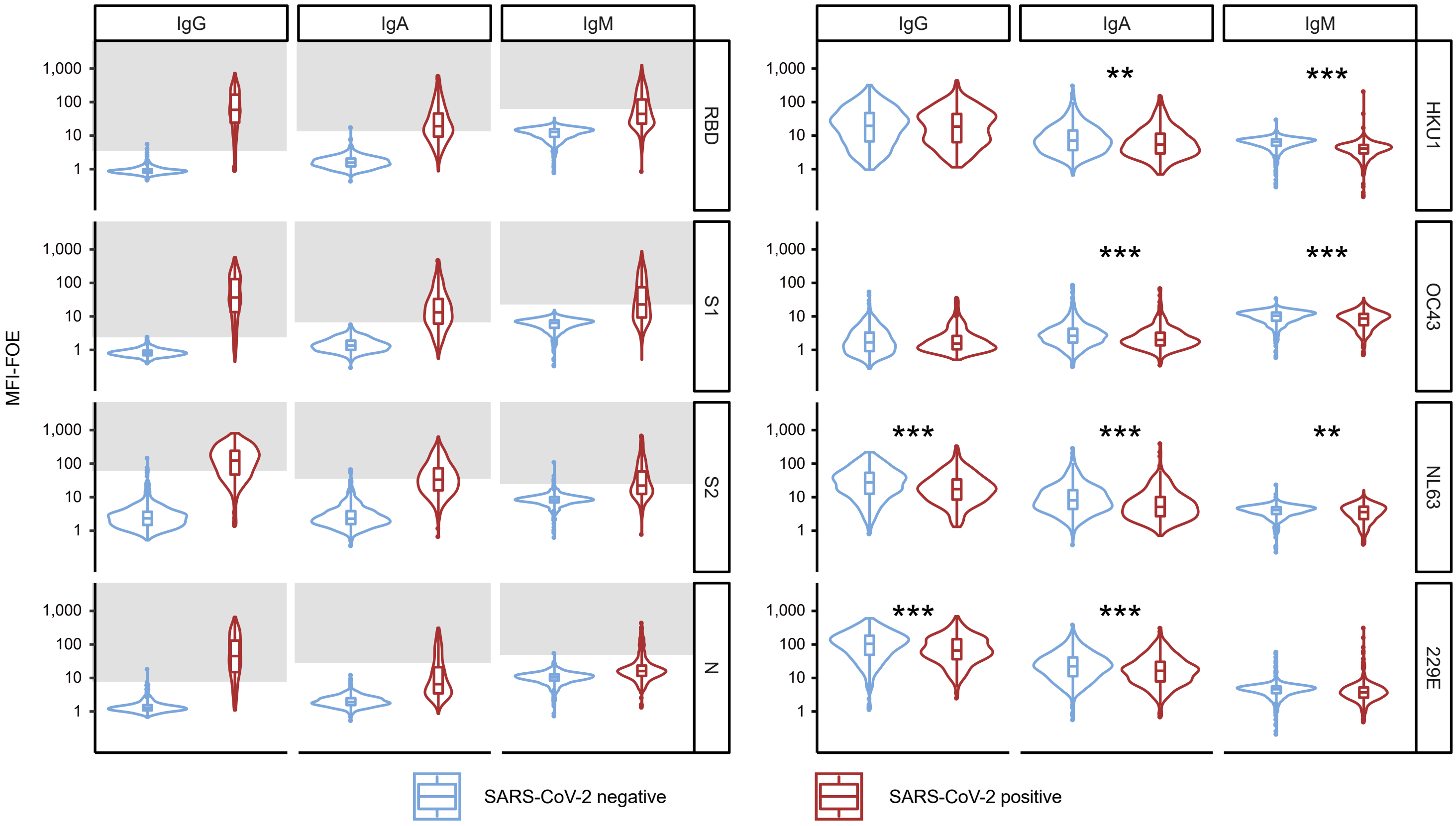

Supplementary Fig. 14. Association between SARS-CoV-2 and HCoV antibody responses. Comparison of ABCORA 5.0 reactivity for SARS-CoV-2 and HCoVs in healthy, SARS-CoV-2 negative and SARS-CoV-2 infected individuals. Healthy donors were sampled in May 2020 (N=653; blue). Plasma from SARS-CoV-2 infected individuals were collected between April 2020 and February 2021 (N=389; red, Training III and Validation VI). Grey boxes indicate values above the individual MFI-FOE cut-offs for SARS-CoV-2 specific responses for each antigen. Stars correspond to levels of significance of two-sided t-tests comparing negative versus positive patients. Levels of significance are corrected by the Bonferroni method for multiple testing and indicated as follows: *$p<0.05/12$, **$p<0.01/12$, ***$p< 0.001/12$ (IgG HKU1: $p=0.74$, IgG OC43: $p=0.75$, IgG NL63: $p=2.2x10^{-07}$, IgG 229E: $p=2.3x10^{-05}$, IgA HKU1: $p=1.9x10^{-04}$, IgA OC43: $p=1.9x10^{-05}$, IgA NL63: $p=2.6x10^{-11}$, IgA 229E: $p=9.2x10^{-07}$, IgM HKU1: $p=3.7x10^{-29}$, IgM OC43: $p=1.5x10^{-06}$, IgM NL63: $p=1.3x10^{-04}$, IgM 229E: $p=4.9x10^{-03}$). Boxplots represent the following: median with the middle line, upper and lower quartiles with the box limits, and 1.5x interquartile ranges with the whiskers.

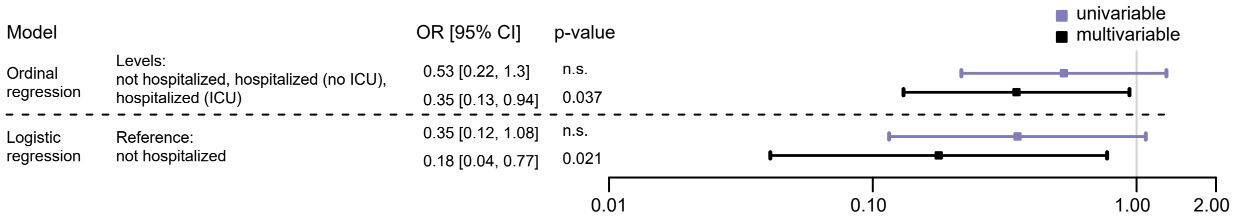

Supplementary Fig. 15. Impact of HCoV immunity on COVID-19 severity. Corresponding analysis to Fig. 8. Association of hospitalization status (not hospitalized (N=16); hospitalized not in ICU (N=42); hospitalized in ICU (N=22)) and HCoV antibody level. Influence of HCoV reactivity (low/high) on the hospitalization status, as estimated with odds ratios, in an ordinal regression (with levels=not hospitalized (N=16); hospitalized not in ICU (N=42); hospitalized in ICU (N=22)) and a logistic regression (reference=not hospitalized (N=16); versus all hospitalized (N=64)). Multivariate analysis is adjusted on age, gender and time since positive RT-PCR. Data is presented as parameter estimation and its 95% confidence interval. Level of significance of the parameter is obtained with a two-sided t-test (p-value is displayed if <0.05, otherwise indicated as n.s.).

Supplementary Table 1. Individual coefficients of variation (LogMFI) per antigen / immunoglobulin[1]					
		RBD	S1	S2	N
IgG	minimum	0.015	0.021	0.024	0.021
	lower quartile	0.050	0.053	0.058	0.043
	median	0.079	0.083	0.085	0.073
	upper quartile	0.10	0.10	0.11	0.093
	maximum	0.12	0.13	0.13	0.11
IgA	minimum	0.026	0.034	0.037	0.035
	lower quartile	0.047	0.053	0.058	0.058
	median	0.078	0.089	0.068	0.072
	upper quartile	0.090	0.094	0.076	0.078
	maximum	0.095	0.10	0.083	0.088
IgM	minimum	0.010	0.014	0.016	0.018
	lower quartile	0.022	0.024	0.027	0.019
	median	0.040	0.039	0.040	0.029
	upper quartile	0.046	0.057	0.049	0.038
	maximum	0.046	0.059	0.051	0.041

[1]Coefficient of variation based on experiments summarized in Supplementary Figure 4.

**Supplementary Table 2. Individual coefficients of variation (LogMFI) per plasma dilution / immunoglobulin**[1]								
		1/100	1/400	1/1,600	1/6,400	1/25,600	1/102,400	1/409,600
IgG	minimum	0.015	0.035	0.064	0.092	0.11	0.073	0.039
	lower quartile	0.018	0.041	0.071	0.1	0.12	0.079	0.049
	median	0.021	0.048	0.080	0.114	0.12	0.087	0.061
	upper quartile	0.023	0.050	0.089	0.124	0.13	0.10	0.071
	maximum	0.024	0.052	0.096	0.128	0.13	0.11	0.079
IgA	minimum	0.034	0.026	0.059	0.078	0.079	0.068	0.062
	lower quartile	0.035	0.030	0.062	0.08	0.083	0.070	0.068
	median	0.036	0.037	0.067	0.083	0.089	0.080	0.081
	upper quartile	0.044	0.046	0.070	0.087	0.097	0.093	0.092
	maximum	0.051	0.054	0.073	0.09	0.10	0.097	0.095
IgM	minimum	0.010	0.022	0.041	0.028	0.019	0.019	0.016
	lower quartile	0.012	0.025	0.043	0.034	0.023	0.020	0.017
	median	0.022	0.033	0.048	0.043	0.036	0.026	0.021
	upper quartile	0.035	0.044	0.054	0.052	0.048	0.033	0.029
	maximum	0.039	0.049	0.057	0.059	0.050	0.035	0.033

[1]Coefficient of variation based on experiments summarized in Supplementary Figure 4.

Supplemetary Table 3. Sensitivity and specificity of ABCORA 2 approaches

			ABCORA 2.0 based on SOC criteria				ABCORA 2.1 logistic regression				ABCORA 2.2 random forest based on ABCORA 2.0 SARS-CoV-2 parameters				ABCORA 2.3 random forest based on ABCORA 2.0 SARS-CoV-2 and HKU1 parameters			
		N	Positive	Negative	Specificity	Sensitivity	Positive	Negative	Specificity	Sensitivity	Positive	Negative	Specificity	Sensitivity	Positive	Negative	Specificity	Sensitivity
Training	I - healthy, prepandemic adults	573	6	567	99.07		5	568	99.23		0	573	100.00		0	573	100.00	
	II - HCoV+ prepandemic adults	75	0	75			0	75			0	75			0	75		
	III - SARS-CoV-2+ adults	175	165	10		94.29	164	11		93.71	175	0		100.00	175	0		100.00
Validation	IV - healthy, prepandemic adults	252	1	251	99.29		1	251	99.52		0	252	99.76		0	252	99.76	
	V - prepandemic children	169	2	167			1	168			1	168			1	168		
	VI - SARS-CoV-2+ adults	214	203	11		94.86	200	14		93.46	204	10		95.33	207	7		96.73
Combined	I + IV - healthy, prepandemic adults	825	7	818			6	819			0	825			0	825		
	II - HCoV+ prepandemic adults	75	0	75	99.16		0	75	99.35		0	75	99.91		0	75	99.91	
	V - prepandemic children	169	2	167			1	168			1	168			1	168		
	III + VI - SARS-CoV-2+ adults	389	368	21		94.60	364	25		93.57	379	10		97.43	382	7		98.20

Suplementary Table 4. ABCORA 2.0 and 5.0 threshold and signal over cut-off settings for ranking individual antigen reactivities positive

		MFI-FOE[1] threshold positive	MFI-FOE threshold borderline[2]	SOC[3] value positive	SOC value borderline
IgG	N	7.7	6.8	> = 1	0.88 < N < 1
	RBD	3.5	2.5	> = 1	0.71 < RBD < 1
	S1	2.3	2	> = 1	0.87 < S1 < 1
	S2	62.8		> = 1	
IgA	N	27.7		> = 1	
	RBD	13.9		> = 1	
	S1	6.7		> = 1	
	S2	35.8		> = 1	
IgM	N	49.2		> = 1	
	RBD	66.8		> = 1	
	S1	22.9		> = 1	
	S2	25.2		> = 1	

[1] FOE = fold over empty beads; FOE cut-offs as depicted in Fig. 1

[2] For IgG responses additional borderline cut-offs for N, RBD, and S1 were defined in order to register lower level reactivity.

[3] SOC = signal over cut-off. SOC values express individual measurements as FOE sample in relation to the respective FOE cut-off.

**Supplementary Table 5. Criteria for definition of SARS-CoV-2 seroconversion status by ABCORA 2.0 and 5.0**		
Seroconversion rating	Description	Measurement criteria
Positive	Full SARS-CoV-2 seroconversion including IgG reactivity	At least 2 measurements > = SOC 1; one of which IgG reactive<br>OR<br>1 measurement > = SOC 1; at least 2 measurements IgG SOC borderline
Positive, partial	Partial SARS-CoV-2 seroconversion with IgA and/or IgM reactiv	At least 2 measurements (IgA and/or IgM > = SOC 1
Negative, weak reactivity	Notable reactivity but below positivity criteria	Single measurements IgG RBD, S1 or NP > = SOC 1<br>OR<br>1 measurement > = SOC 1 combined with 1 measurement IgG SOC borderline
Negative, indetermina	Isolated reactivity	Single measurements (IgG S2, IgA RBD, S1, S2 or N, IgM RBD, S1, S2 or N) > = SOC 1
Negative	No SARS-CoV-2 reactivity	No measurement > = SOC 1

Supplementary Table 6. Median of sensitivity and specificity of ABCORA 2.3 approach in the 5-fold cross validation

	ABCORA 2.3	
	Specificity	Sensitivity
Training	100.00	100.00
Validation	99.50	96.20
Combined	99.75	98.10

Supplementary Table 7. Sensitivity and specificity assessment with the Anti-SARS-CoV-2 Verification Panel for Serology Assays (NIBSC 20/B770)				
Assay system	Target antigen	Ig subtype	Sensitivity	Specificity
ABCORA 2.0	RBD, S1, S2, N	IgG, IgA, IgM	100.00%	100.00%
ABCORA 2.1			100.00%	100.00%
ABCORA 2.2			100.00%	100.00%
ABCORA 2.3			100.00%	100.00%
Abbott Architect SARS-CoV-2 IgG	N	IgG	95.65%	100.00%
DiaPro COVID-19 IgG	Spike, N	IgG	100.00%	100.00%
DiaPro COVID-19 IgM	not published	IgM	82.61%	100.00%
DiaSorin SARS-CoV-2 IgM	not published	IgM	65.22%	100.00%
EUROIMMUN Anti-SARS-CoV-2 ELISA (IgA)	S1	IgA	100.00%	85.71%
EUROIMMUN Anti-SARS-CoV-2 ELISA (IgG)	S1	IgG	100.00%	100.00%
EUROIMMUN Anti-SARS-CoV-2 NCP ELISA (IgG)	N	IgG	100.00%	100.00%
Fortress COVID-19 IgM	not published	IgM	73.91%	100.00%
Fortress COVID-19 Total Antibody	not published	Total Ig	100.00%	85.71%
Liaison SARS-CoV-2 S1/S2 IgG	S1, S2	IgG	100.00%	92.86%
PHE Colindale Anti-SARS-CoV-2 ELISA in-house	not published	not published	100.00%	100.00%
Roche Elecsys Anti-SARS-CoV-2	N	Total Ig	100.00%	100.00%
Siemens SARS-CoV-2 IgG	RBD	IgG	91.30%	100.00%
Siemens SARS-CoV-2 Total Antibody	RBD	IgG, IgM	100.00%	100.00%

**Supplementary Table 8. Sensitivity of ABCORA compared to commercial serology tests**			
Assay system	SARS-CoV-2+ patients (N=171)		Sensitivity
ABCORA 2.0	positive	162	94.74%
	negative	9	
ABCORA 2.3	positive	171	100.00%
	negative	0	
EUROIMMUN Anti-SARS-CoV-2 ELISA IgG assay	positive	153	89.47%
	negative	18	
Roche Elecsys Anti-SARS-CoV-2 (S1) assay	positive	160	93.57%
	negative	11	
Roche Elecsys Anti-SARS-CoV-2 (N) assay	positive	157	91.81%
	negative	14	

Supplementary Table 9. Sensitivity and specificity of different ULR-S1-SOC models depending on different composite S1 SOC and NT50 thresholds

NT50 threshold for defining neutralizers groups	Composite S1 SOC value threshold	Specificity	Sensitivity
100	6	84.00	80.00
	10.5	95.00	69.00
250	9.7	81.00	81.00
	17.3	94.00	67.00

Supplementary Table 10. Sensitivity and specificity of the ABCORA 5 approaches

			ABCORA 5.0<br>based on ABCORA 2.0 SOC criteria		ABCORA 5.2<br>Random forest defined in ABCORA 2.2		ABCORA 5.3<br>Random forest defined in ABCORA 2.3		ABCORA 5.4<br>Random forest trained on ABCORA 5.0 SARS-CoV-2 data		ABCORA 5.5<br>Radom forest trained on ABCORA 5.0 SARS-CoV-2 and HCoV data	
		N	Specificity	Sensitivity	Specificity	Sensitivity	Specificity	Sensitivity	Specificity	Sensitivity	Specificity	Sensitivity
Training	I - healthy, prepandemic adults	573	99.69		99.38		99.69		100.00		100.00	
	II - HCoV+ prepandemic adults	75										
	III - SARS-CoV-2+ adults	175		95.43		96.57		96.57		100.00		100.00
Validation	IV - healthy, prepandemic adults	252	99.05		98.57		98.81		98.81		99.52	
	V - prepandemic children	169										
	VI - SARS-CoV-2+ adults	214		96.26		96.26		96.73		97.20		97.66
Combined	I + IV - healthy, prepandemic adults	825										
	II - HCoV+ prepandemic adults	75	99.44		99.06		99.35		99.53		99.81	
	V - prepandemic children	170										
	III + VI - SARS-CoV-2+ adults	389		95.89		96.40		96.66		98.46		98.71

Supplementary Table 11. Gender and age distribution per cohort

		Gender			Age		Days since positive RT-PCR		Days since symptom onset		Hospitalization status			
		Man, N (%)	Woman, N (%)	Data unavailable, N (%)	Median (min, max)	Data unavailable, N (%)	Median (min, max)	Data unavailable, N (%)	Median (min, max)	Data unavailable, N (%)	Not hospitalized, N (%)	Hospitalized no ICU, N (%)	Hospitalized ICU, N (%)	Data unavailable, N (%)
SARS-CoV-2 positive adults	Training (III), N=175	128 (73.1%)	47 (26.9%)	0	50.5 (11, 95)	1 (0.6%)	23 (0,194)	30 (17.1%)	33 (3,198)	33 (18.9%)	61 (34.9%)	44 (25.1%)	22 (12.6%)	48 (27.4%)
	Validation (VI), N=214	170 (79.4%)	36 (16.8%)	8 (3.7%)	45.5 (16, 92)	28 (13.1%)	62 (0,331)	79 (36.9%)	85.5 (29,335)	112 (52.3%)	33 (15.4%)	0	0	181 (84.6%)
Healthy, prepandemic adults	Training (I), N=573	337 (58.8%)	204 (35.6%)	32 (5.6%)	50 (19, 76)	32 (5.6%)								
	Validation (IV), N=252	155 (61.5%)	97 (38.5%)	0	42 (19, 75)	0								
Recent hCoV infection, prepandemic adults	Training (II), N=75	48 (64.0%)	27 (36.0%)	0	59 (22, 78)	0								
Prepandemic children	Validation (V), N=169	96 (56.8%)	73 (42.9%)	0	8.5 (0,18)	0								
Healthy pandemic adults	N=672	390 (58.0%)	282 (42.0%)	0	40.0 (18, 75)	0								
Total	N=2130	1324 (62.2%)	766 (36.0%)	40 (1.9%)	43.0 (0, 95)	61 (2.9%)								

Supplementary Table 12. CoV-derived antigens					
Antigen	Origin	Tag	Expression host	Manucfacturer	Catalog number
NP	SARS-CoV-2	C-terminal polyhistidine tag	Baculovirus-Insect cells	Sino Biological Europe GmbH, Eschborn, Germany	50488-V08B
RBD	SARS-CoV-2	C-terminal polyhistidine tag	HEK293 cells		40592-V08H
S1 subunit	SARS-CoV-2	C-terminal polyhistidine tag	HEK293 cells		40591-V08H
S2 subunit	SARS-CoV-2	C-terminal polyhistidine tag	Baculovirus-Insect cells		40590-V08B
S1 subunit	hCoV-HKU1	C-terminal polyhistidine tag	HEK293 cells		40021-V08H
S1 subunit	hCoV-OC43	C-terminal polyhistidine tag	HEK293 cells		40607-V08H1
S1 subunit	hCoV-NL63	C-terminal polyhistidine tag	HEK293 cells		40600-V08H
S1 subunit	hCoV-229E	C-terminal polyhistidine tag	HEK293 cells		40601-V08H

Supplementary Table 13. Origin and characteristics of antibody reagents

Monclonal / Polyclonal	Isotype	Conjugate	Supplier	Clone	Catalog number	Stock concentration	Starting dilution in study	Application in study
SARS-CoV-2 (2019-nCoV) Spike S1 monoclonal antibody	Rabbit IgG	-	Sino Biological Europe GmbH, Eschborn, Germany	007	40150-R007	n.a.	1/100	Primary antibody to control antigen coupling to beads
SARS-CoV / SARS-CoV-2 Nucleoprotein / NP polyclonal antibody	Rabbit IgG	-	Sino Biological Europe GmbH, Eschborn, Germany	polyclonal	40143-T62	n.a.	1/100	Primary antibody to control antigen coupling to beads
Anti-SARS-CoV-2 Spike Glycoprotein S1 monoclonal antibody [CR3022]	Human IgG1	-	Abcam, Cambridge, UK	CR3022	ab273073	1 mg/ml	1/25	Quantification of SARS-CoV-2 specific antibodies in plasma
Recombinant Anti-SARS spike glycoprotein monoclonal antibody [CR3022]	Human IgA	-	Abcam, Cambridge, UK	CR3022	ab278112	1 mg/ml	1/25	Quantification of SARS-CoV-2 specific antibodies in plasma
Recombinant Anti-SARS spike glycoprotein monoclonal antibody [CR3022]	Human IgM	-	Abcam, Cambridge, UK	CR3022	ab278111	1 mg/ml	1/25	Quantification of SARS-CoV-2 specific antibodies in plasma
Anti-His tag monoclonal antibody	Mouse IgG	-	Sino Biological Europe GmbH, Eschborn, Germany	02	105327-MM02T	5 mg/ml		Coupling of His-tagged antigens to beads
Anti-human IgG Fc monoclonal antibody	Mouse IgG	PE	BioLegend, San Diego, CA	HP6017	409304	0.2 mg/ml	1/500	Secondary antibody for ABCORA
Anti-human IgA	Goat IgG	PE	Southern Biotech, Birmingham, AL	polyclonal	2050-09	0.5 mg/ml	1/500	Secondary antibody for ABCORA
Anti-human IgM	Goat IgG	PE	Southern Biotech, Birmingham, AL	polyclonal	2020-09	0.5 mg/ml	1/500	Secondary antibody for ABCORA
Anti-mouse IgG	Goat IgG	PE	BioLegend, San Diego, CA	polyclonal	405307	0.2 mg/ml	1/20	Secondary antibody to control anti-His antibody loading
Anti-rabbit IgG	Goat IgG	PE	Southern Biotech, Birmingham, AL	polyclonal	4030-09	0.5 mg/ml	1/500	Secondary antibody to control antigen coupling
Anti-SARS-CoV-2 Verification Panel for Serology Assays	Plasma	-	NIBSC, Potters Bar, UK		20/B770			Verification of ABCORA assay
First WHO International Standard Anti-SARS-CoV-2 Immunoglobulin (Human)	Plasma pool	-	NIBSC, Potters Bar, UK		20/136	1000 IU/ml		Quantification of SARS-CoV-2 specific antibodies in plasma
