# Supplementary material for: Multifactorial seroprofiling dissects the contribution of pre-existing human coronaviruses responses to SARS-CoV-2 immunity
Source: Nat Commun. 2021 Nov 18;12:6703. doi: 10.1038/s41467-021-27040-x (PMC8602384; doi:10.1038/s41467-021-27040-x)
Supplement: Supplementary file 3 — Reporting Summary [file 41467_2021_27040_MOESM3_ESM.pdf]

nature research

Corresponding author(s): Alexandra Trkola

Last updated by author(s): Oct 28, 2021

Reporting Summary

Nature Research wishes to improve the reproducibility of the work that we publish. This form provides structure for consistency and transparency in reporting. For further information on Nature Research policies, see our Editorial Policies and the Editorial Policy Checklist.

Statistics

For all statistical analyses, confirm that the following items are present in the figure legend, table legend, main text, or Methods section.

n/a	Confirmed	
☐	☒	The exact sample size (*n*) for each experimental group/condition, given as a discrete number and unit of measurement
☐	☒	A statement on whether measurements were taken from distinct samples or whether the same sample was measured repeatedly
☐	☒	The statistical test(s) used AND whether they are one- or two-sided<br>*Only common tests should be described solely by name; describe more complex techniques in the Methods section.*
☐	☒	A description of all covariates tested
☐	☒	A description of any assumptions or corrections, such as tests of normality and adjustment for multiple comparisons
☐	☒	A full description of the statistical parameters including central tendency (e.g. means) or other basic estimates (e.g. regression coefficient) AND variation (e.g. standard deviation) or associated estimates of uncertainty (e.g. confidence intervals)
☐	☒	For null hypothesis testing, the test statistic (e.g. *F*, *t*, *r*) with confidence intervals, effect sizes, degrees of freedom and *P* value noted<br>*Give P values as exact values whenever suitable.*
☒	☐	For Bayesian analysis, information on the choice of priors and Markov chain Monte Carlo settings
☒	☐	For hierarchical and complex designs, identification of the appropriate level for tests and full reporting of outcomes
☐	☒	Estimates of effect sizes (e.g. Cohen's *d*, Pearson's *r*), indicating how they were calculated

Our web collection on statistics for biologists contains articles on many of the points above.

Software and code

Policy information about availability of computer code

Data collection	Data management was performed in R (Version 3.6.3) and GraphPad Prism Version 9.0.0)
Data analysis	Statistical analyses were performed in R (Version 3.6.3) and GraphPad Prism (Version 9.0.0). The following R packages were used: ggplot2 (version 3.3.3), randomForest (version 4.6.14), ranger (version 0.12.1), lmer (version 1.1.27), lmerTest (version 3.1.3) ComplexHeatmap (version 2.6.2).<br>Codes to assess serostatus based on the ABCORA 2.3 method are available at: https://github.com/chlpasin/SARS-CoV-2-serology

For manuscripts utilizing custom algorithms or software that are central to the research but not yet described in published literature, software must be made available to editors and reviewers. We strongly encourage code deposition in a community repository (e.g. GitHub). See the Nature Research guidelines for submitting code & software for further information.

Data

Policy information about availability of data

All manuscripts must include a data availability statement. This statement should provide the following information, where applicable:

- Accession codes, unique identifiers, or web links for publicly available datasets
- A list of figures that have associated raw data
- A description of any restrictions on data availability

Serological measurements from all patient cohorts are made available as excel file in Supplementary Data 2. Codes to assess serostatus based on the ABCORA 2.3 method are available at: https://github.com/chlpasin/SARS-CoV-2-serology.
Demographic and clinical information are not publicly available due to ethic restrictions.

Field-specific reporting

Please select the one below that is the best fit for your research. If you are not sure, read the appropriate sections before making your selection.

- [x] Life sciences
- [ ] Behavioural & social sciences
- [ ] Ecological, evolutionary & environmental sciences

For a reference copy of the document with all sections, see nature.com/documents/nr-reporting-summary-flat.pdf

Life sciences study design

All studies must disclose on these points even when the disclosure is negative.

Sample size	No sample size was determined. Samples were obtained based on availability in routine testing or biobanks.
Data exclusions	No data were excluded
Replication	All ABCORA measurements to SARS-CoV-2 and HCoV antigens in patients cohorts, were analyzed with quality controls included on every plate (see also material and methods section). Due to the large number of samples no replicates were done.<br>Neutralization measurements were done in duplicates.<br>Establishment titration experiments were done in at least 2 independent replicates. Assay variability was assessed with 31 independent titrations.
Randomization	Allocation was not random. Statistical analyses assessing interdependencies between HCoVs and SARS-CoV-2 were adjusted on age, gender and time since positive RT-PCR or time since symptoms onset.
Blinding	Investigators who conducted the SARS-COV-2 antibody experiments had no information on patient demographics, neutralization activity and SARS-CoV-2 infection status at the time of analysis.<br>As clinical data was used from routine diagnostic, no blinding was possible for clinical staff collecting blood samples.

Reporting for specific materials, systems and methods

We require information from authors about some types of materials, experimental systems and methods used in many studies. Here, indicate whether each material, system or method listed is relevant to your study. If you are not sure if a list item applies to your research, read the appropriate section before selecting a response.

Materials & experimental systems

n/a	Involved in the study	
☐	☒	Antibodies
☐	☒	Eukaryotic cell lines
☒	☐	Palaeontology and archaeology
☒	☐	Animals and other organisms
☐	☒	Human research participants
☐	☒	Clinical data
☒	☐	Dual use research of concern

Methods

n/a	Involved in the study	
☒	☐	ChIP-seq
☒	☐	Flow cytometry
☒	☐	MRI-based neuroimaging

Antibodies

Antibodies used	Details of antibodies used in this study are provided in Supplementary Table 13.
Validation	SARS-CoV-2 (2019-nCoV) Spike S1 Antibody (SinoBiological, 40150-R007) was validated by the company for ELISA.<br>SARS-CoV / SARS-CoV-2 Nucleoprotein / NP Antibody (SinoBiological, 40143-T62) was validated by the company for ELISA and Western Blot applications.<br>Anti-SARS-CoV-2 Spike Glycoprotein antibody [CR3022] (Abcam, ab273073) was validated by the company for indirect ELISA and ELISA at an assay dependent concentration.<br>Anti-SARS-CoV-2 Spike Glycoprotein antibody [CR3022] (Abcam, ab278112) was validated by the company for ELISA at an assay dependent concentration.<br>Anti-SARS-CoV-2 Spike Glycoprotein antibody [CR3022] (Abcam, ab278111) was validated by the company for ELISA at concentration of 0.005-10 ug/ml.<br>Anti-His tag monoclonal antibody (SinoBiological, 105327-MM02T) was validated by the company for WB applications at a dilution of 1/1000-1/10000.<br>First WHO International Standard Anti-SARSCoV-2 Immunoglobulin (Human) (NIBSC, 20/136) was assessed by Mattiuzzo et al. Establishment of the WHO International Standard and Reference Panel for anti-SARS-CoV-2 antibody. 2020, WHO Expert Committee on Biological Standardization. WHO/BS/2020.2403.

Eukaryotic cell lines

Policy information about cell lines

Cell line source(s)	293-T cells were obtained from the American Type Culture Collection (ATCC CRL-11268). HT1080/ACE2cl.14 cells 31 were kindly provided by P. Bieniasz, Rockefeller University, NY.
Authentication	None of the cell lines used were authenticated again after reception from the specified original source.
Mycoplasma contamination	In the Trkola laboratory, cell lines are routinely tested for mycoplasma contamination. No such contamination was detected in the cells used for the present study.
Commonly misidentified lines (See ICLAC register)	No commonly misidentified cell lines were used in the study.

Human research participants

Policy information about studies involving human research participants

Population characteristics	When available, the following covariates were considered: gender (63% men, 36% women, 2% not available), age (median 43 years-old, 3% not available), days since positive RT-PCR (median 62 days, 37% not available), days since symptom onset (median 86 days, 53% not available), and hospitalization status (24% not hospitalized, 11% hospitalized no ICU, 6% hospitalized ICU, 59% not available).
Recruitment	No patient enrollment was conducted for the present study, all specimen were either available through leftover samples from routine diagnostic testing or provided from biobanks of approved studies.
Ethics oversight	All experiments involving samples from human donors were conducted with the approval of the responsible local ethics committee (Kantonale Ethikkommission) Zurich, Switzerland (BASEC Nrs 2020-01327, 2020-00363; 2021-00437; 2020-00787), in accordance with the provisions of the Declaration of Helsinki and the Good Clinical Practice guidelines of the International Conference on Harmonisation. Samples were obtained from the following sources: i) Zurich blood donation services (ZHBDS): Anonymized healthy adult plasma from pre-pandemic time points (January 2019, May 2019 and January 2020) and from the first wave of the pandemic in Zurich, Switzerland (May 2020) were provided by the ZHBDS internal serum repository and consent for this study was waived by the ethics committee (BASEC 2021-00437). ii) Anonymized leftover specimens from routine diagnostics at the Institute of Medical Virology, University of Zurich, the University Children Hospital Zurich and the Cantonal Hospital Winterthur (BASEC Nrs 2020-01327, 2021-00437). Written informed consent was obtained from all participants whose sample was taken during the pandemic at the University Hospital Zurich (BASEC 2020-01327). For pandemic samples from other hospitals and pre-pandemic samples consent was waived by the ethics committee. iii) Healthcare workers with RT-PCR confirmed SARS-CoV-2 infection participating in a study at the University Hospital Zurich (BASEC 2020-00363). Written informed consent was obtained from all participants. iv) Male plasma donors participating in a SARS-CoV-2 plasma therapy study conducted at the University Hospital Zurich (CPT-ZHP, Swissmedic 2020TpP1004; BASEC 2020-00787). Written informed consent for research was obtained from all participants.

Note that full information on the approval of the study protocol must also be provided in the manuscript.

Clinical data

Policy information about clinical studies

All manuscripts should comply with the ICMJEguidelines for publication of clinical research and a completedCONSORT checklist must be included with all submissions.

Clinical trial registration	The CPT Study is registered in Swissmedic 2020TpP1004 and swissethics BASEC 2020 -00363
Study protocol	https://www.kofam.ch/en/snctp-portal/search/118861/study/50562
Data collection	SARS-CoV-2 positive samples from diagnostic repositories (Institute of Medical Virology, UZH, Cantonal Hospital Winterthur): Samples were collected starting March 2020 until March 2021.<br>Healthy adult plasma were collected from pre-pandemic time points (January 2019, May 2019 and January 2020) and from the first wave of the pandemic in Zurich, Switzerland (May 2020) and were provided by the ZHBDS internal serum repository.<br>Samples from healthcare workers with RT-PCR confirmed SARS-CoV-2 infection participating in a study conducted at the University Hospital Zurich were collected during the first wave in April and May 2020.
Outcomes	Only the CPT study was a clinical trial, but the clinical outcomes were defined for CPT recipients, and we analyzed data from CPT donors only. Therefore no clinical outcome was relevant here.<br>All other studies are not clinical trials.
