# Supplementary material for: Multifactorial seroprofiling dissects the contribution of pre-existing human coronaviruses responses to SARS-CoV-2 immunity
Source: Nat Commun. 2021 Nov 18;12:6703. doi: 10.1038/s41467-021-27040-x (PMC8602384; doi:10.1038/s41467-021-27040-x)
Supplement: Supplementary file 4 — Description of Additional Supplementary Files [file 41467_2021_27040_MOESM4_ESM.pdf]

Multifactorial seroprofiling dissects the contribution of pre-existing human coronaviruses responses to SARS-CoV-2 immunity

Description of Additional Supplementary Files

File name: Supplementary Data 1
Description: Individual LogMFI-FOE, AUC, EC50, CR3022 and WHO Standard quantification corresponding to Figure 2.

File name: Supplementary Data 2
Description: Serological measurements generated with the ABCORA assay from all patients described in Supplementary Table 11.
